# 5-Cyclo­hexyl-4-methyl-1*H*-pyrazol-3(2*H*)-one monohydrate

**DOI:** 10.1107/S1600536810039164

**Published:** 2010-10-09

**Authors:** Tara Shahani, Hoong-Kun Fun, R. Venkat Ragavan, V. Vijayakumar, S. Sarveswari

**Affiliations:** a X-ray Crystallography Unit, School of Physics, Universiti Sains Malaysia, 11800 USM, Penang, Malaysia; b Organic Chemistry Division, School of Advanced Sciences, VIT University, Vellore 632 014, India

## Abstract

In the title compound, C_10_H_16_N_2_O·H_2_O, the cyclo­hexane ring is in a chair conformation and its least-squares plane makes a dihedral angle of 53.68 (5)° with the approximately planar pyrazole ring [maximum deviation = 0.034 (1) Å]. Pairs of inter­molecular N—H⋯O hydrogen bonds form inversion dimers between neighbouring pyrazolone mol­ecules, generating *R*_2_^2^(8) ring motifs. The pyrazolone and water mol­ecules are further linked by inter­molecular N—H⋯O, C—H⋯O and O—H⋯O hydrogen bonds into two-dimensional sheets parallel to the *bc* plane.

## Related literature

For pyrazole derivatives and their microbial activities, see: Ragavan *et al.* (2009 ▶, 2010 ▶). For related structures, see: Shahani *et al.* (2009 ▶, 2010*a* ▶,*b* ▶,*c* ▶). For ring conformations, see: Cremer & Pople (1975 ▶). For hydrogen-bond motifs, see: Bernstein *et al.* (1995 ▶). For bond-length data, see: Allen *et al.* (1987 ▶). For the stability of the temperature controller used for the data collection, see: Cosier & Glazer (1986 ▶).
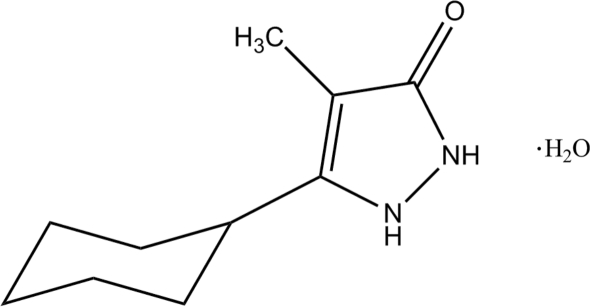


## Experimental

### 

#### Crystal data

C_10_H_16_N_2_O·H_2_O
*M* *_r_* = 198.26
Monoclinic, 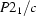

*a* = 13.4959 (3) Å
*b* = 6.2497 (1) Å
*c* = 13.9268 (3) Å
β = 112.782 (1)°
*V* = 1083.02 (4) Å^3^
*Z* = 4
Mo *K*α radiation
μ = 0.09 mm^−1^
*T* = 100 K
0.46 × 0.27 × 0.23 mm

#### Data collection

Bruker SMART APEXII CCD area-detector diffractometer
Absorption correction: multi-scan (*SADABS*; Bruker, 2009 ▶) *T*_min_ = 0.962, *T*_max_ = 0.981
26403 measured reflections
4715 independent reflections
3863 reflections with *I* > 2σ(*I*)
*R*_int_ = 0.033

#### Refinement

*R*[*F* ^2^ > 2σ(*F* ^2^)] = 0.042
*wR*(*F* ^2^) = 0.117
*S* = 1.03
4715 reflections
199 parameters
All H-atom parameters refined
Δρ_max_ = 0.55 e Å^−3^
Δρ_min_ = −0.28 e Å^−3^

### 

Data collection: *APEX2* (Bruker, 2009 ▶); cell refinement: *SAINT* (Bruker, 2009 ▶); data reduction: *SAINT*; program(s) used to solve structure: *SHELXTL* (Sheldrick, 2008 ▶); program(s) used to refine structure: *SHELXTL*; molecular graphics: *SHELXTL*; software used to prepare material for publication: *SHELXTL* and *PLATON* (Spek, 2009 ▶).

## Supplementary Material

Crystal structure: contains datablocks global, I. DOI: 10.1107/S1600536810039164/is2608sup1.cif

Structure factors: contains datablocks I. DOI: 10.1107/S1600536810039164/is2608Isup2.hkl

Additional supplementary materials:  crystallographic information; 3D view; checkCIF report

## Figures and Tables

**Table 1:** Hydrogen-bond geometry (Å, °)

*D*—H⋯*A*	*D*—H	H⋯*A*	*D*⋯*A*	*D*—H⋯*A*
N1—H1*N*1⋯O1*W*^i^	0.889 (14)	1.866 (14)	2.7513 (9)	173.7 (12)
N2—H1*N*2⋯O1^ii^	0.924 (14)	1.842 (13)	2.7552 (9)	169.5 (13)
O1*W*—H1*W*1⋯O1	0.889 (17)	1.851 (17)	2.7354 (8)	173.2 (18)
O1*W*—H1*W*2⋯O1^iii^	0.860 (19)	1.961 (19)	2.8007 (9)	165.0 (16)
C5—H5*A*⋯O1*W*^i^	0.987 (14)	2.503 (15)	3.4161 (12)	153.7 (11)

Symmetry codes: (i) 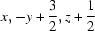; (ii) 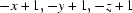; (iii) 
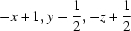
.

**Fig. 1.**
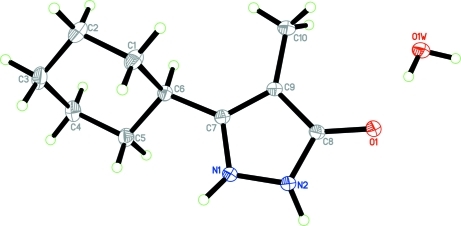
The molecular structure of the title compound, showing 50% probability displacement ellipsoids and the atom numbering scheme.

**Fig. 2.**
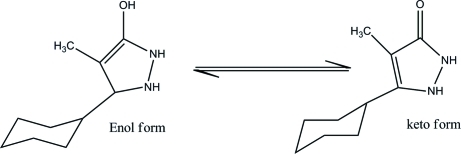
Enol-to-keto tautomerism of the title compound during crystallization process.

**Fig. 3.**
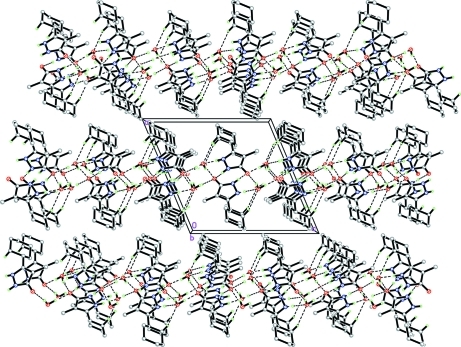
The crystal packing of the title compound, viewed two-dimensional arrays parallel to the bc plane. Dashed lines indicate hydrogen bonds.

## supplementary crystallographic information

### Comment

Antibacterial and antifungal activities of the azoles are most widely studied and some of them are used in clinical practice as anti-microbial agents. However, the existence of azole-resistant strains had led to the development of new antimicrobial compounds. In particular, pyrazole derivatives are also extensively studied and used as antimicrobial agents. Pyrazole is an important class of heterocyclic compound and many pyrazole derivatives are reported to have the broad spectrum of biological activities, such as anti-inflammatory, antifungal, herbicidal, anti-tumour, cytotoxic and antiviral activities. Pyrazole derivatives also act as antiangiogenic agents, A3 adenosine receptor antagonists, neuropeptide YY5 receptor antagonists, kinase inhibitor for treatment of type-2 diabetes, hyperlipidemia, obesity, and thrombopiotinmimetics. Recently urea derivatives of pyrazoles have been reported as potent inhibitors of p38 kinase. Since the high electronegativity of halogens (particularly chlorine and fluorine) in the aromatic part of the drug molecules play an important role in enhancing their biological activity, we are interested to have 4-fluoro or 4-chloro substitution in the aryls of 1,5-diaryl pyrazoles. As part of our on-going research aiming on the synthesis of new antimicrobial compounds, we have reported the synthesis of novel pyrazole derivatives and their microbial activities (Ragavan *et al.*, 2009, 2010).

The asymmetric unit of the title compound, (Fig. 1), consists of one 5-cyclohexyl-4-methyl-1*H*-pyrazol-3(2*H*)-one molecule (C1—C10/N1/N2/O1) and one water molecule. The 3-cyclohexyl-4-methyl-1 *H*-pyrazol-5-ol undergoes an enol-to-keto tautomerism during the crystallization process (Fig. 2). The cyclohexane ring is in a chair conformation with puckering parameters of Q = 0.5813 (10) Å, Θ = 177.06 (10)° and φ = 164.9 (19)° (Cremer & Pople, 1975), and its least-squares plane is at an angle of 53.68 (5)° with the approximately planar pyrazole ring (C7–C9/N1/N2; maximum deviation of 0.034 (1) Å at atom N2). The bond lengths (Allen *et al.*, 1987) and angles are within normal ranges and comparable to those closely related structures (Shahani *et al.*, 2009, 2010*a*,*b*,*c*)

In the crystal packing (Fig. 3), pairs of intermolecular N2—H1N2···O1 hydrogen bonds (Table 1) form dimers with neighbouring molecules, generating *R*_2_^2^(8) ring motifs (Bernstein *et al.*, 1995). The molecules are further linked by intermolecular N1—H1N1···O1W, C5—H5A···O1W, O1W—H1W1···O1 and O1W—H1W2 ···O1 hydrogen bonds (Table 1) into two-dimensional sheets parallel to the *bc* plane.

### Experimental

The compound has been synthesized using the method available in the literature (Ragavan *et al.*, 2010) and recrystallized using the ethanol-chloroform 1:1 mixture. Yield: 77%. *m.p.*: 205.4–206.2 °C.

### Refinement

All H atoms were located in a difference fourier map and were refined freely [refined distances: N—H = 0.889 (14)–0.923 (14) Å, C—H = 0.93 (12)–1.022 (13) Å and O—H = 0.858 (18)–0.888 (17) Å].

### Crystal data

**Table:**

C_10_H_16_N_2_O·H_2_O	*F*(000) = 432
*M**_r_* = 198.26	*D*_x_ = 1.216 Mg m^−^^3^
Monoclinic, *P*2_1_/*c*	Mo *K*α radiation, λ = 0.71073 Å
Hall symbol: -P 2ybc	Cell parameters from 9296 reflections
*a* = 13.4959 (3) Å	θ = 3.0–34.7°
*b* = 6.2497 (1) Å	µ = 0.09 mm^−^^1^
*c* = 13.9268 (3) Å	*T* = 100 K
β = 112.782 (1)°	Block, colourless
*V* = 1083.02 (4) Å^3^	0.46 × 0.27 × 0.23 mm
*Z* = 4	

### Data collection

**Table:**

Bruker SMART APEXII CCD area-detector diffractometer	4715 independent reflections
Radiation source: fine-focus sealed tube	3863 reflections with *I* > 2σ(*I*)
graphite	*R*_int_ = 0.033
φ and ω scans	θ_max_ = 35.0°, θ_min_ = 1.6°
Absorption correction: multi-scan (*SADABS*; Bruker, 2009)	*h* = −20→21
*T*_min_ = 0.962, *T*_max_ = 0.981	*k* = −10→10
26403 measured reflections	*l* = −21→21

### Refinement

**Table:**

Refinement on *F*^2^	Primary atom site location: structure-invariant direct methods
Least-squares matrix: full	Secondary atom site location: difference Fourier map
*R*[*F*^2^ > 2σ(*F*^2^)] = 0.042	Hydrogen site location: inferred from neighbouring sites
*wR*(*F*^2^) = 0.117	All H-atom parameters refined
*S* = 1.03	*w* = 1/[σ^2^(*F*_o_^2^) + (0.0623*P*)^2^ + 0.2027*P*] where *P* = (*F*_o_^2^ + 2*F*_c_^2^)/3
4715 reflections	(Δ/σ)_max_ < 0.001
199 parameters	Δρ_max_ = 0.55 e Å^−^^3^
0 restraints	Δρ_min_ = −0.28 e Å^−^^3^

### Special details

**Table:**

Experimental. The crystal was placed in the cold stream of an Oxford Cyrosystems Cobra open-flow nitrogen cryostat (Cosier & Glazer, 1986) operating at 100.0 (1) K.
Geometry. All e.s.d.'s (except the e.s.d. in the dihedral angle between two l.s. planes) are estimated using the full covariance matrix. The cell e.s.d.'s are taken into account individually in the estimation of e.s.d.'s in distances, angles and torsion angles; correlations between e.s.d.'s in cell parameters are only used when they are defined by crystal symmetry. An approximate (isotropic) treatment of cell e.s.d.'s is used for estimating e.s.d.'s involving l.s. planes.
Refinement. Refinement of *F*^2^ against ALL reflections. The weighted *R*-factor *wR* and goodness of fit *S* are based on *F*^2^, conventional *R*-factors *R* are based on *F*, with *F* set to zero for negative *F*^2^. The threshold expression of *F*^2^ > σ(*F*^2^) is used only for calculating *R*-factors(gt) *etc*. and is not relevant to the choice of reflections for refinement. *R*-factors based on *F*^2^ are statistically about twice as large as those based on *F*, and *R*- factors based on ALL data will be even larger.

### Fractional atomic coordinates and isotropic or equivalent isotropic displacement parameters (Å^2^)

**Table:**

	*x*	*y*	*z*	*U*_iso_*/*U*_eq_	
O1	0.47692 (5)	0.58283 (9)	0.36443 (4)	0.01649 (11)	
N1	0.35298 (5)	0.86770 (11)	0.49923 (5)	0.01570 (12)	
N2	0.43455 (5)	0.74179 (11)	0.49437 (5)	0.01560 (12)	
C1	0.22589 (7)	1.31323 (13)	0.36124 (7)	0.02077 (15)	
C2	0.12483 (7)	1.45257 (14)	0.33028 (7)	0.02375 (16)	
C3	0.07738 (7)	1.44773 (14)	0.41354 (7)	0.02298 (16)	
C4	0.05310 (7)	1.21880 (14)	0.43594 (7)	0.02146 (16)	
C5	0.15219 (7)	1.07543 (13)	0.46434 (7)	0.01902 (14)	
C6	0.19853 (6)	1.08190 (12)	0.37959 (6)	0.01492 (13)	
C7	0.29215 (6)	0.93410 (11)	0.40136 (5)	0.01425 (13)	
C8	0.41832 (6)	0.71084 (12)	0.39255 (5)	0.01420 (13)	
C9	0.32907 (6)	0.83939 (12)	0.33169 (5)	0.01507 (13)	
C10	0.28323 (7)	0.85575 (15)	0.21552 (6)	0.02220 (16)	
O1W	0.39076 (5)	0.38748 (10)	0.17409 (5)	0.02017 (12)	
H1A	0.2820 (10)	1.3696 (19)	0.4284 (10)	0.026 (3)*	
H1B	0.2551 (10)	1.319 (2)	0.3063 (11)	0.030 (3)*	
H2A	0.0700 (11)	1.398 (2)	0.2637 (11)	0.029 (3)*	
H2B	0.1410 (11)	1.602 (2)	0.3181 (11)	0.033 (3)*	
H3A	0.1301 (11)	1.516 (2)	0.4797 (10)	0.028 (3)*	
H3B	0.0083 (10)	1.536 (2)	0.3909 (10)	0.027 (3)*	
H4A	−0.0056 (10)	1.161 (2)	0.3741 (11)	0.029 (3)*	
H4B	0.0270 (10)	1.214 (2)	0.4932 (10)	0.028 (3)*	
H5A	0.2079 (11)	1.124 (2)	0.5307 (11)	0.032 (3)*	
H5B	0.1334 (10)	0.922 (2)	0.4749 (10)	0.025 (3)*	
H6	0.1416 (10)	1.028 (2)	0.3127 (9)	0.020 (3)*	
H10A	0.2285 (15)	0.956 (3)	0.1918 (15)	0.066 (5)*	
H10B	0.3351 (14)	0.891 (3)	0.1877 (13)	0.053 (5)*	
H10C	0.2553 (13)	0.719 (3)	0.1795 (14)	0.060 (5)*	
H1N1	0.3647 (11)	0.938 (2)	0.5581 (11)	0.033 (3)*	
H1N2	0.4682 (11)	0.646 (2)	0.5476 (11)	0.034 (3)*	
H1W1	0.4222 (13)	0.442 (3)	0.2377 (13)	0.046 (4)*	
H1W2	0.4323 (13)	0.284 (3)	0.1729 (13)	0.052 (5)*	

### Atomic displacement parameters (Å^2^)

**Table:**

	*U*^11^	*U*^22^	*U*^33^	*U*^12^	*U*^13^	*U*^23^
O1	0.0200 (3)	0.0165 (2)	0.0143 (2)	0.00467 (19)	0.00818 (19)	0.00135 (18)
N1	0.0190 (3)	0.0166 (3)	0.0112 (2)	0.0047 (2)	0.0057 (2)	0.0004 (2)
N2	0.0187 (3)	0.0162 (3)	0.0116 (3)	0.0051 (2)	0.0056 (2)	0.0017 (2)
C1	0.0210 (3)	0.0161 (3)	0.0261 (4)	0.0025 (3)	0.0102 (3)	0.0048 (3)
C2	0.0251 (4)	0.0165 (3)	0.0282 (4)	0.0053 (3)	0.0088 (3)	0.0047 (3)
C3	0.0227 (4)	0.0170 (3)	0.0272 (4)	0.0040 (3)	0.0074 (3)	−0.0051 (3)
C4	0.0197 (3)	0.0206 (4)	0.0257 (4)	0.0018 (3)	0.0105 (3)	−0.0039 (3)
C5	0.0208 (3)	0.0179 (3)	0.0211 (3)	0.0028 (3)	0.0113 (3)	0.0010 (3)
C6	0.0158 (3)	0.0139 (3)	0.0145 (3)	0.0018 (2)	0.0053 (2)	−0.0002 (2)
C7	0.0166 (3)	0.0135 (3)	0.0122 (3)	0.0015 (2)	0.0052 (2)	0.0010 (2)
C8	0.0173 (3)	0.0138 (3)	0.0118 (3)	0.0012 (2)	0.0060 (2)	0.0009 (2)
C9	0.0180 (3)	0.0156 (3)	0.0112 (3)	0.0034 (2)	0.0052 (2)	0.0015 (2)
C10	0.0275 (4)	0.0259 (4)	0.0118 (3)	0.0087 (3)	0.0059 (3)	0.0024 (3)
O1W	0.0246 (3)	0.0216 (3)	0.0139 (2)	0.0035 (2)	0.0071 (2)	0.0007 (2)

### Geometric parameters (Å, °)

**Table:**

O1—C8	1.2880 (9)	C4—C5	1.5290 (11)
N1—C7	1.3555 (9)	C4—H4A	0.985 (14)
N1—N2	1.3760 (9)	C4—H4B	0.989 (13)
N1—H1N1	0.889 (14)	C5—C6	1.5354 (10)
N2—C8	1.3622 (9)	C5—H5A	0.986 (14)
N2—H1N2	0.923 (14)	C5—H5B	1.020 (13)
C1—C2	1.5326 (12)	C6—C7	1.4982 (10)
C1—C6	1.5378 (11)	C6—H6	1.009 (12)
C1—H1A	1.012 (13)	C7—C9	1.3836 (10)
C1—H1B	0.987 (13)	C8—C9	1.4226 (10)
C2—C3	1.5267 (13)	C9—C10	1.4953 (11)
C2—H2A	0.995 (14)	C10—H10A	0.93 (2)
C2—H2B	0.986 (14)	C10—H10B	0.948 (17)
C3—C4	1.5267 (13)	C10—H10C	0.987 (19)
C3—H3A	1.013 (14)	O1W—H1W1	0.888 (17)
C3—H3B	1.022 (13)	O1W—H1W2	0.858 (18)
			
C7—N1—N2	108.07 (6)	H4A—C4—H4B	106.1 (11)
C7—N1—H1N1	126.7 (9)	C4—C5—C6	111.20 (7)
N2—N1—H1N1	118.0 (9)	C4—C5—H5A	109.6 (8)
C8—N2—N1	108.86 (6)	C6—C5—H5A	108.8 (8)
C8—N2—H1N2	125.1 (9)	C4—C5—H5B	110.4 (7)
N1—N2—H1N2	119.0 (8)	C6—C5—H5B	109.5 (7)
C2—C1—C6	109.64 (7)	H5A—C5—H5B	107.3 (11)
C2—C1—H1A	109.2 (7)	C7—C6—C5	113.01 (6)
C6—C1—H1A	108.4 (7)	C7—C6—C1	112.05 (6)
C2—C1—H1B	110.0 (8)	C5—C6—C1	110.36 (6)
C6—C1—H1B	110.7 (8)	C7—C6—H6	105.2 (7)
H1A—C1—H1B	108.9 (10)	C5—C6—H6	108.0 (7)
C3—C2—C1	111.37 (7)	C1—C6—H6	107.9 (7)
C3—C2—H2A	108.8 (8)	N1—C7—C9	109.38 (6)
C1—C2—H2A	109.1 (8)	N1—C7—C6	121.81 (6)
C3—C2—H2B	109.6 (8)	C9—C7—C6	128.78 (7)
C1—C2—H2B	110.7 (8)	O1—C8—N2	122.31 (7)
H2A—C2—H2B	107.2 (11)	O1—C8—C9	130.36 (6)
C2—C3—C4	111.10 (7)	N2—C8—C9	107.32 (6)
C2—C3—H3A	109.2 (7)	C7—C9—C8	105.99 (6)
C4—C3—H3A	109.7 (8)	C7—C9—C10	128.25 (7)
C2—C3—H3B	110.8 (7)	C8—C9—C10	125.68 (7)
C4—C3—H3B	109.0 (8)	C9—C10—H10A	111.8 (12)
H3A—C3—H3B	106.9 (11)	C9—C10—H10B	113.4 (10)
C3—C4—C5	111.51 (7)	H10A—C10—H10B	108.0 (15)
C3—C4—H4A	109.1 (8)	C9—C10—H10C	114.0 (11)
C5—C4—H4A	109.8 (8)	H10A—C10—H10C	108.0 (15)
C3—C4—H4B	111.4 (8)	H10B—C10—H10C	101.0 (14)
C5—C4—H4B	108.8 (8)	H1W1—O1W—H1W2	104.0 (14)
			
C7—N1—N2—C8	−6.34 (8)	C5—C6—C7—C9	154.47 (8)
C6—C1—C2—C3	57.93 (10)	C1—C6—C7—C9	−80.10 (10)
C1—C2—C3—C4	−56.21 (10)	N1—N2—C8—O1	−173.49 (7)
C2—C3—C4—C5	54.34 (10)	N1—N2—C8—C9	5.81 (8)
C3—C4—C5—C6	−54.98 (9)	N1—C7—C9—C8	−0.75 (9)
C4—C5—C6—C7	−176.77 (7)	C6—C7—C9—C8	−178.70 (7)
C4—C5—C6—C1	56.89 (9)	N1—C7—C9—C10	176.30 (8)
C2—C1—C6—C7	175.19 (7)	C6—C7—C9—C10	−1.65 (14)
C2—C1—C6—C5	−57.94 (9)	O1—C8—C9—C7	176.10 (8)
N2—N1—C7—C9	4.32 (9)	N2—C8—C9—C7	−3.12 (8)
N2—N1—C7—C6	−177.56 (6)	O1—C8—C9—C10	−1.05 (14)
C5—C6—C7—N1	−23.26 (10)	N2—C8—C9—C10	179.74 (8)
C1—C6—C7—N1	102.18 (8)		

### Hydrogen-bond geometry (Å, °)

**Table:**

*D*—H···*A*	*D*—H	H···*A*	*D*···*A*	*D*—H···*A*
N1—H1N1···O1W^i^	0.889 (14)	1.866 (14)	2.7513 (9)	173.7 (12)
N2—H1N2···O1^ii^	0.924 (14)	1.842 (13)	2.7552 (9)	169.5 (13)
O1W—H1W1···O1	0.889 (17)	1.851 (17)	2.7354 (8)	173.2 (18)
O1W—H1W2···O1^iii^	0.860 (19)	1.961 (19)	2.8007 (9)	165.0 (16)
C5—H5A···O1W^i^	0.987 (14)	2.503 (15)	3.4161 (12)	153.7 (11)

Symmetry codes: (i) *x*, −*y*+3/2, *z*+1/2; (ii) −*x*+1, −*y*+1, −*z*+1; (iii) −*x*+1, *y*−1/2, −*z*+1/2.
